# High-Strength Low-Alloy Steels for Automobiles: Microstructure and Mechanical Properties

**DOI:** 10.3390/ma18204660

**Published:** 2025-10-10

**Authors:** Guoqiang Ma, Bo Gao, Zhen Chen, Yuquan Li, Ruirui Wu, Hailian Gui, Zhibing Chu

**Affiliations:** 1 College of Materials Science and Engineering, Taiyuan University of Science and Technology, Taiyuan 030024, China; gaobo@tyust.edu.cn (B.G.); s202314210388@stu.tyust.edu.cn (Y.L.); wrr1126@tyust.edu.cn (R.W.); guihailian@tyust.edu.cn (H.G.); 2 Jiangsu Belight Laboratory, State Key Laboratory of Advanced Casting Technologies, Nanjing University of Science and Technology, Nanjing 210094, China; zchen@njust.edu.cn

**Keywords:** high-strength low-alloy steel, microstructure, mechanical properties, strengthening mechanism, Hall–Petch relationship

## Abstract

High-strength low-alloy (HSLA) steel is widely used in automotive industry for reduction of consumption and emissions. The microstructure and mechanical properties of two automotive HSLA steels with different strength grades were systematically investigated in present study. Microstructural characterization was conducted using optical microscopy (OM), scanning electron microscopy (SEM), and electron backscatter diffraction (EBSD), while mechanical properties were evaluated with Vickers hardness tester and tensile tests. Both steels exhibited a ferrite matrix with spheroidized carbides/pearlites. However, Sample A displayed equiaxed ferrite grains with localized pearlite colonies, while Sample B featured pronounced elongated ferrite grains with a band structure. Tensile testing revealed that Sample B had higher ultimate tensile stress and yield stress compared to Sample A. Texture analysis indicated that both steels were dominated by α-fiber and γ-fiber textures, with minor θ-fiber texture, resulting in minimal mechanical anisotropy between the rolling direction (RD) and transverse direction (TD). The quantitative assessment of strengthening mechanisms, based on microstructural parameters and experimental data, revealed that grain boundary strengthening dominates, with dislocation strengthening also contributing significantly. This work provides the first comprehensive quantification of individual strengthening contributions in automotive HSLA steels, offering critical guidance for developing further higher-strength automotive steels.

## 1. Introduction

Vehicle manufacturers are under increasing pressure to decrease fuel consumption and greenhouse gas emissions, while improving safety standards. A proven strategy to achieve both reduced consumption and emissions is to lighten the vehicles [1,2,3]. Studies show that a 10% reduction in vehicle weight can lead to a 6–8% fuel economy improvement and 4–6% carbon emissions reduction. To make cars lighter while maintaining or even enhancing their strength to meet safety standards, manufacturers can utilize materials with a high strength-to-weight ratio, such as higher strength steels, and minimize material use where possible. Therefore, the research and development of high-strength automotive steel has consistently been a key focus within the automotive industry. Currently, automotive steels are classified into five primary groups: mild steels, conventional high-strength steels (HSS), and advanced high-strength steels (AHSS, including the first to third generation) [4]. Higher-strength steels offer the potential to reduce vehicle weight by using thinner sheets, while maintaining or even enhancing crash performance [5]. However, the use of thinner and stronger sheets presents several challenges, including limited formability, high costs of alloying elements, sheet springback, and die wear, etc. [6,7]. Consequently, HSS remain the predominant material choice for automotive steel, particularly for structural components that require substantial material volumes, such as the automotive body frame, automobile chassis frame, and subframe.

High-strength low-alloy (HSLA) steel is a type of HSS. It provides ensures sufficient strength and has excellent cold stamping performance and weldability, facilitating processing and manufacturing. HSLA steel is mainly used in key structural components that require high strength and account for a relatively large proportion of a vehicle’s weight. HSLA steels are C-Mn steels strengthened through micro-alloying with a very small amount of Ti, V, or Nb. These steels have a yield stress of 220–850 MPa and an ultimate tensile stress of 340–1000 MPa [4,8,9,10,11,12]. HSLA steel for automobiles is a high value-added steel, and its chemical composition and manufacturing processes are crucial as they significantly affect the steel’s microstructure and mechanical properties. For example, Nb and Ti are known for their grain refinement and precipitation strengthening capabilities, while Mo enhances hardenability and high-temperature strength.

Precipitation strengthening is a key mechanism for enhancing the strength of HSLA steels, achieved through the formation of fine, dispersed second-phase particles. Chen et al. [13] investigated the effects of Ti, Ti-Mo, and Ti-Nb microalloy additions on precipitation strengthening in HSLA steels, finding that (Ti, Mo)C carbides maintain nanometer sizes and contribute most to hardness due to their superior thermal stability during continuous and interrupted cooling. Park et al. [14] examined how Mo and W additions affect precipitation hardening in Ti and Nb-microalloyed HSLA steels, revealing that the Nb-Mo combination yields the highest precipitation hardening due to distinct microstructural evolution. Militzer et al. [15] revealed that increased Nb content reduces recrystallization rates, enhances retained strain accumulation, and influences austenite decomposition and precipitation during cooling. Furthermore, Vervynckt et al. [16] extensively investigated the precipitation behavior of Nb in HSLA steel during various austenite processing stages, revealing that recrystallization occurs during precipitate nucleation and coarsening but is suppressed during precipitation growth due to Zener pinning effects. Show et al. [17] modified the composition of DMR-249A HSLA steel by adding V, with or without Ti, to produce plates that met stringent yield strength and toughness requirements, attributing the success to nanoscale V-rich carbonitride particles. Nitrogen exhibits a strong affinity with Ti, Nb, and V, which drives the formation of stable carbonitride precipitates. These carbonitride precipitates are more effective in precipitation strengthening compared to carbide. The nitrogen content significantly influences the content, size, chemical composition, and coarsening tendency of these precipitates. Thermodynamic models are therefore employed to calculate the chemical composition of austenite and the content of the formed carbonitride precipitates [18,19].

In terms of tailoring the matrix microstructure, Lambert-Perlade et al. [20] studied the austenite-to-bainite transformation in low alloy structural steel post simulated welding heat treatment, revealing upper bainite packet formation and the role of variant pairs in limiting austenite plastic strain, thereby promoting bainite growth during cooling. The addition of B is particularly effective in suppressing pearlite formation and broadening the cooling range for bainitic transformations [21]. Similarly, Kim et al. [22] examined the effects of rolling temperature on the microstructure and mechanical properties of Ti-Mo microalloyed hot-rolled steel, finding that rolling through non-recrystallisation zones enhances strength and impact toughness through fine grain refinement, dislocation development, and dense carbide precipitation.

The mechanical properties of HSLA steels are significantly influenced by the manufacturing processes, especially thermomechanical controlled processing (TMCP). Advanced high-strength steels for automotive applications have been designed with a carbide-free bainitic microstructure through TMCP and continuous annealing, achieving superior uniform elongation, formability, and a balanced strength-ductility ratio compared to commercial steels [23]. Zhao et al. [24] derived regression equations linking TMCP parameters (rolling and cooling temperatures, cooling rate) to mechanical properties, identifying optimal conditions for achieving superior strength and toughness through acicular ferrite microstructures. Vervynckt et al. [25] focused on optimizing TMCP in low-alloyed steels, examining the fundamental mechanisms controlling the non-recrystallisation temperature (T-nr) and discussing methods for its accurate determination in industrial rolling processes. Additionally, advanced processing techniques such as Wire and Arc Additive Manufacturing (WAAM) have been explored for HSLA steels, revealing that varying heat inputs affect cooling rates but not microstructural constituents, resulting in nearly isotropic mechanical properties with excellent ductility and strength [26].

Numerous studies have systematically expounded the relationship between microstructural quantitative parameters and mechanical properties [27,28,29,30,31,32,33,34]. Based on the principle of linearly additive contributions of various strengthening mechanisms, the flow stress was calculated considering the contributions from solid-solution strengthening (σ_ss_), grain boundary strengthening (based on the Hall–Petch equation), dislocation density strengthening (Taylor strengthening), and particle strengthening, etc. However, in many previous studies, the Hall–Petch coefficients (K values) and the contributions of solid-solution strengthening exhibit significant variations owing to differences in chemical composition, manufacturing processes, and microstructures [33,35,36,37,38,39]. We need to systematically investigate the strengthening mechanisms of automotive HSLA steels and quantify the contributions of each factor, with the aim of providing guidance for the development of even higher-strength automotive steels. Therefore, this study aims to investigate two typical automotive steels, that is, hot-rolled pickling plate QSTE340TM and QSTE500TM produced by Compact Strip Production (CSP) [9,10,11,12]. We explored the influence of microstructure on the mechanical properties of these steels by analyzing their chemical compositions and microstructural characteristics and quantifying these parameters with the previously established linearly additive equation. Moreover, we assessed the relative contribution of each strengthening mechanism in these steels, thus offering valuable guidance for the production and processing of automotive steels.

## 2. Materials and Methods

The starting steel sheets were two types of automotive HSLA steels with different strength grades, QSTE340TM and QSTE500TM (taken from Hunan Valin Lianyuan Steel, Loudi, China, and hereafter referred to as Sample A and Sample B, respectively), produced by CSP processes. These steels are hot-rolled pickling sheets widely used in automotive structural components [9,10,11,12]. For Sample A, the finisher entry and delivery temperatures during hot rolling are controlled at 1200 °C and 890 °C, respectively, with a coiling temperature of 620 °C. For Sample B, the finisher entry temperature was set at 1245 °C, while the other temperature parameters remained the same as those for Sample A. The final thicknesses of both steel sheets was 2 mm, and all samples were prepared near the typical 1/4 width position of the sheets. The detailed parameters were shown in Table 1.

The chemical composition of the materials was measured using an optical emission spectrometer (ARL8860, manufactured by Thermo Fisher Scientific, Waltham, MA, USA). Samples with dimensions of 30 mm × 30 mm × 2 mm were cut from the sheets. Their surfaces were ground from coarse to 320 to 2000 grit abrasive papers. To minimize measurement errors, each sample was measured three times, and the average values were shown in Table 2.

Microstructural observations were conducted using optical microscopy (OM), scanning electron microscopy (SEM), and electron backscatter diffraction (EBSD). The OM used is a Zeiss Axio Vert.A1 (manufactured by ZEISS, Oberkochen, Germany). The SEM employed is a Zeiss GeminiSEM 500 field-emission scanning electron microscope (FE-SEM, manufactured by ZEISS, Oberkochen, Germany). EBSD analysis was conducted using an Oxford AZtec EBSD system (Symmetry version) equipped on the SEM. For OM and SEM, samples were ground from 320 to 2000 grit, mechanically polished, and then etched for approximately 30 s in a solution of nitric acid and ethyl alcohol (4:96 by volume). EBSD samples were ground with 2000-grit abrasive paper and then electropolished in a mixture of perchloric acid and ethyl alcohol (1:9 by volume) at −20 °C under a voltage of 20 V for 1 min. Microstructural analysis was performed on the rolling plane, encompassing both the rolling direction (RD) and transverse direction (TD).

**Figure 1:**
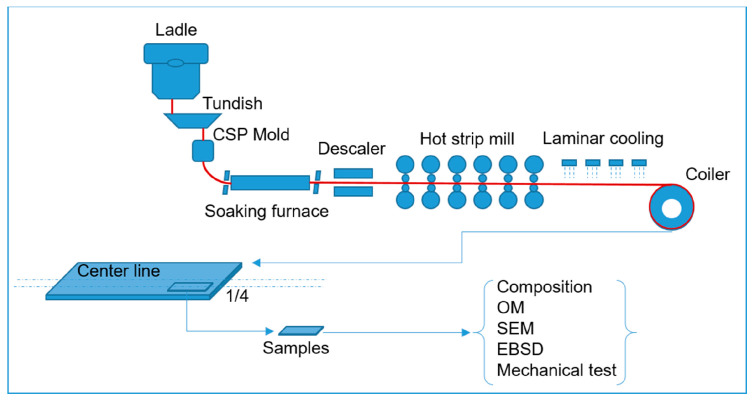
Methodology framework of present study.

Microhardness measurements were carried out using an MH-3 Vickers hardness tester (manufactured by Shanghai Honc Instrument Technology, Shanghai, China) with an applied load of 500 g for 10 s. To assess hardness variation across the material thickness, test points were uniformly distributed throughout the sheet’s thickness. Tensile tests were performed using an MTS Exceed E45 electronic universal testing system (manufactured by MTS Systems Corporation, Eden Prairie, MN, USA), with real-time strain data collected via a mechanical extensometer featuring a 50 mm gauge length. Dog bone-shaped tensile specimens were cut from the sheets, with a gauge length of 50 mm, width of 12.5 mm, and thickness of 2 mm (matching the original sheet thickness). Tests were conducted at a constant strain rate of 1 mm/min, with at least three specimens tested per condition to ensure accuracy. The yield stress was determined based on the stress corresponding to the lower yield point. The investigation steps, presented in the form of a methodology framework, are shown in Figure 1.

## 3. Results and Discussion

**Figure 2:**
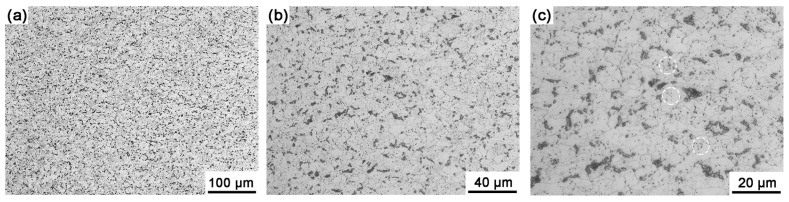
Optical micrographs of Sample A with different magnifications show that equiaxed polygonal ferrite grains with spheroidized carbides/pearlites dispersed within the grain boundaries and matrix. (**a**) low-magnification micrograph, (**b**) medium-magnification micrograph, (**c**) high-magnification micrograph. The spheroidized carbides/pearlites were marked in the dotted circles.

The metallurgical microstructure of Sample A is shown in Figure 2. Based on its chemical composition, the steady-state microstructure should consist of ferrite and a minor volume of pearlite. Observations revealed that the actual microstructure of Steel A comprises predominantly ferrite along with spheroidized carbides/pearlites (marked in Figure 2c). The pearlite colonies were sparse and primarily localized along the ferrite grain boundaries. Meanwhile, a portion of the carbides undergoes spheroidization during hot rolling and subsequent coiling processes, dispersing within the ferrite matrix. Etching revealed a microstructure dominated by equiaxed polygonal ferrite grains.

**Figure 3:**
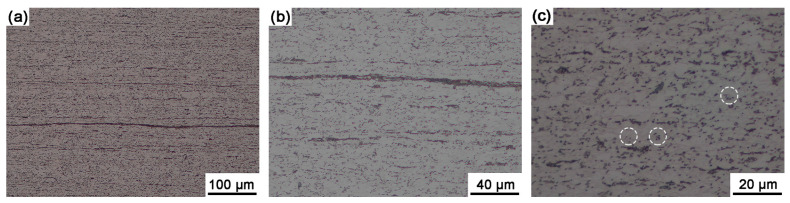
Optical micrographs of Sample B with different magnifications show that elongated ferrite grains with typical band structure formed by hot rolling, retained in the matrix. (**a**) low-magnification micrograph, (**b**) medium-magnification micrograph, (**c**) high-magnification micrograph. The spheroidized carbides/pearlites were marked in the dotted circles.

The metallurgical microstructure of Sample B also consists of a ferrite matrix with spheroidized carbides/pearlites (Figure 3). However, pearlite colonies were barely observable, while the carbides exhibited fragmentation and uniform spheroidization, dispersing homogeneously within the matrix. Under the influence of hot rolling deformation, the microstructure developed a pronounced elongated morphology and formed a typical band structure [7]. The spheroidized carbide/pearlite particles (marked in Figure 3c) were dispersed within the grains, and the ferrite grains exhibited an elongated polygonal shape, reflecting the combined effects of dynamic recovery and static recrystallization during cooling.

**Figure 4:**
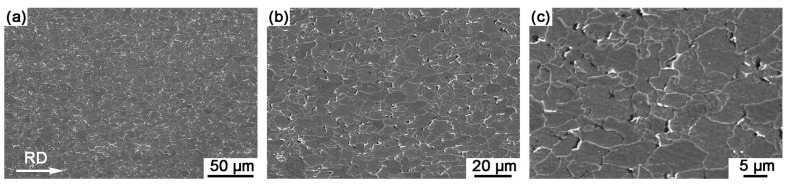
SEM observations of Sample A with different magnifications. (**a**) overall structure at low magnification, (**b**) detailed features at medium magnification, (**c**) microstructure at high magnification.

The refined microstructure of Sample A is clearly revealed in Figure 4. After chemical etching, the ferrite grain boundaries were fully exposed, with a small amount of residual pearlite forming islands adjacent to these boundaries. During hot rolling, the high-temperature austenite undergoes significant plastic deformation along the rolling direction, forming a typical lamellar deformation structure. Subsequently, during coiling at 620 °C, the ferrite transformed into equiaxed polygonal grains through static recrystallization, while cementite partially dissolves at this temperature. During subsequent cooling, the remaining cementite undergoes spheroidization and coarsening, though its spheroidization degree is still significantly lower than that achieved by bell annealing [7]. Finally, partial pearlite degenerated during thermal deformation, resulting in a characteristic microstructure where spheroidized carbides particles dispersed within the ferrite matrix.

**Figure 5:**
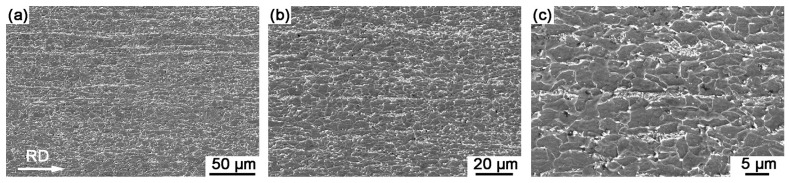
SEM observations of Sample B with different magnifications. (**a**) overall structure at low magnification, (**b**) detailed features at medium magnification, (**c**) microstructure at high magnification.

The SEM microstructure of Sample B is presented in Figure 5. Compared to Sample A, Sample B exhibited more pronounced spheroidization of carbides, primarily attributed to its higher carbon content, which facilitated greater dissolution of carbides and subsequent enhanced nucleation and growth during cooling. Additionally, the typical band structure, characterized by alternating bands of ferrite and carbides/pearlites aligned along the rolling direction, with elongated ferrite grains being particularly evident. SEM observations clearly reveal the grain boundaries in both experimental steels. To determine the average grain diameter, a total of 100 grains per steel sample were measured using Nanomeasurer software 1.2.5 for statistical analysis of grain diameters in random directions based on SEM observations. The results showed that grain size ranges from 1.4 μm to 14.5 μm for Sample A and from 0.9 μm to 10.1 μm for Sample B, with average grain sizes of 5.16 ± 2.77 μm and 3.53 ± 1.65 μm, respectively.

**Figure 6:**
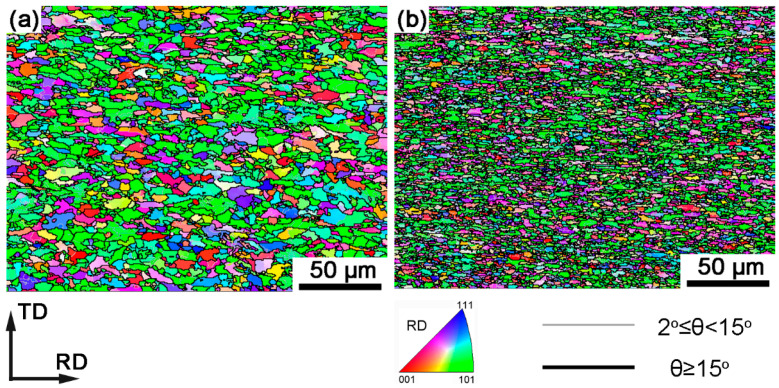
EBSD inverse pole figure (IPF) maps of the two experimental steels: (**a**) Sample A, (**b**) Sample B.

Figure 6 shows the EBSD inverse pole figure (IPF) maps of the two experimental steels. The grains exhibit a typical polygonal shape. The grain size of Sample A is significantly larger than that of Sample B, which further corroborates the statistical results obtained from SEM analysis. Specifically, the average grain sizes are 5.16 ± 2.77 μm for Sample A and 3.53 ± 1.65 μm for Sample B, respectively. The IPF maps reveal a preferred grain orientation, for example, with a pronounced α-fiber texture (<110>//RD). Therefore, further orientation distribution function (ODF) analysis was conducted to quantify the texture intensity and distribution.

**Figure 7:**
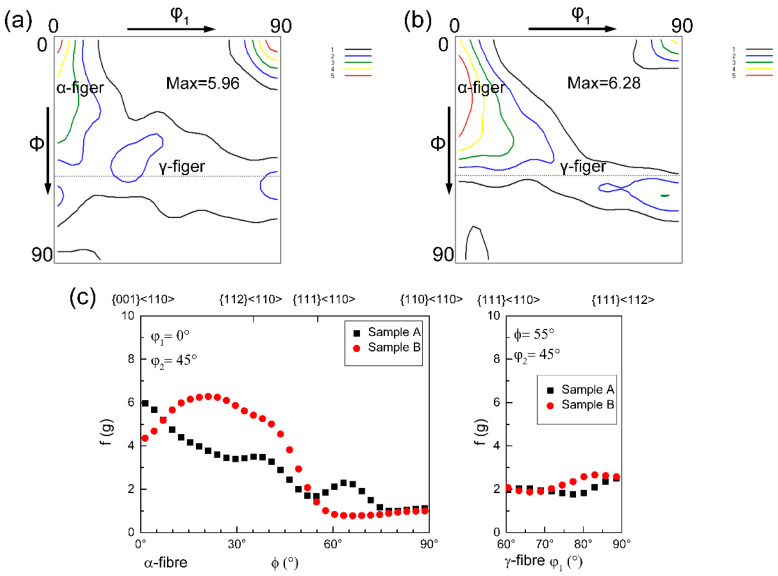
Orientation distribution functions (φ_2_ = 45° sections) revealing texture evolution for the two experimental steels: (**a**) Sample A, (**b**) Sample B, and (**c**) orientation intensity along the α-fiber and γ-fiber.

Since the two experimental steels exhibit a body-centered cubic (bcc) lattice at room temperature, their primary textures were analyzed on φ_2_ = 45° sections of the Euler space [40]. The results revealed the coexistence of α-fiber and γ-fiber (<111>//ND) textures in both steels, accompanied by a minor presence of θ-fiber texture (<100>//ND) (Figure 7). As seen in Figure 7c, Steel B showed a significantly stronger α-fiber texture compared to Steel A, while their γ-fiber textures exhibited similar and relatively low intensities. The texture characteristics significantly influence the mechanical anisotropy of the materials, as reflected by the variations in Taylor factor (M) values across different texture components.

**Figure 8:**
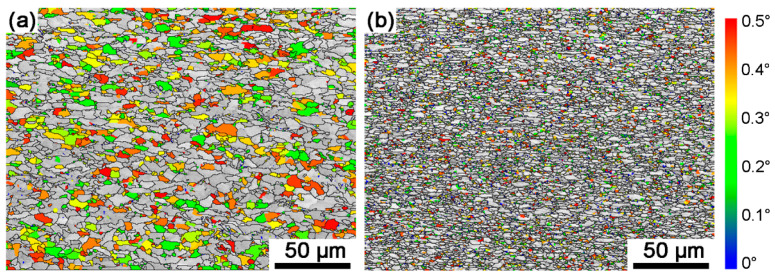
Grain boundary maps and grain state maps of the two experimental steels: (**a**) Sample A, (**b**) Sample B. In the map, black lines represent boundaries larger than 15°. Grains with an interior misorientation angle of less than 0.5° are identified as recrystallized grains and are colored according to the corresponding color legend inset. The remaining grains are marked in gray.

The two steels were produced with hot rolling, with a final rolling temperature of 890 °C, during which dynamic recrystallization and dynamic recovery occurred. Sample A exhibited more pronounced dynamic recrystallization. In contrast, Sample B experienced significant pinning effects, which suppressed recrystallization and retained a deformed grain structure. The suppression of recrystallization in Sample B can be attributed to the presence of Nb, which is the most effective micro-additive employed to elevate the recrystallization temperature. The combined suppression of recrystallization during cooling and phase transformation refinement ensured a refined grain structure, with grains elongated along the rolling direction. As shown in Figure 8, the grain state analysis revealed that Sample A contained a substantial fraction of recrystallized grains, while Sample B exhibited finer grain sizes with a higher proportion of elongated deformed/subgrain structures. Accordingly, the two steels displayed distinct dislocation densities within their grains, requiring consideration of these differences in dislocation density for their subsequent strengthening mechanisms.

**Figure 9:**
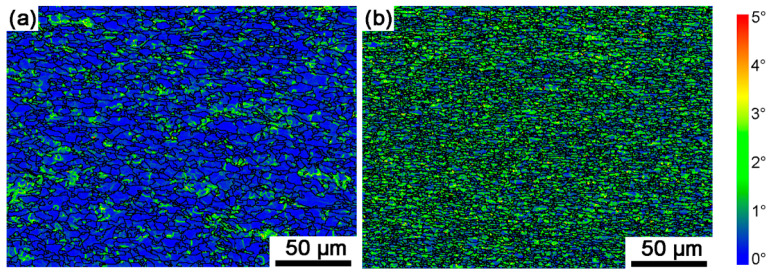
Kernel average misorientation (KAM) maps of the two experimental steels: (**a**) Sample A, (**b**) Sample B.

Kernel average misorientation (KAM) maps display the distribution of local crystallographic misorientations, calculated as the mean orientation difference between a reference point and its neighbors. It can be applied to compute dislocation density [41,42]. In Figure 9, the kernel average misorientation is calculated using a 5 × 5 kernel with an upper threshold taken as 5° [34]. KAM values exhibit significant regional heterogeneity, with elevated regions corresponding to dislocation accumulation zones. The average kernel average misorientation values (KAM_ave_) were calculated using [43]:

(1) $${KAM}_{ave}=exp[\frac{1}{N}\sum_{1}^{i}ln{KAM}_{L,i}]$$

where the N is the total data number, and KAM_L,i_ is the local misorientation for every point detected in maps. The calculated KAM_ave_ results are 0.47° and 1.07° for Sample A and Sample B, respectively.

The geometrically necessary dislocation density (ρ^GND^) can be calculated from EBSD data [41,42], and the ρ^GND^ was determined using the following equation [42]:

(2) $$\rho^{GND}=a\theta /\mu b$$

where a is a constant (taken as 2), θ is local misorientation equal to KAM_ave_, μ is the step size (0.5 μm) used for EBSD data collection, and b is the Burgers vector magnitude (0.248 nm for bcc structure of Fe). Hughes et al. [44] demonstrated that grain boundaries in deformed metals are predominantly populated by non-redundant dislocations. Thus, the ρ^GND^ can be assumed the same approximately with total dislocation density during hot rolling. The calculate results of ρ^GND^ are 1.32 × 10^14^ and 3.01 × 10^14^ for Sample A and Sample B, respectively. The dislocation strengthening was calculated as equation [32,45]:

(3) $$\sigma_{dis}=M\alpha bG{\left(\rho^{GND}\right)}^{1/2}$$

where M = 2.9 for the observed mixture of α-fibers and γ-fibers in bcc, α is a constant taken to be 0.166 [32,33]. The calculated results are 107.0 MPa and 161.6 MPa for Sample A and Sample B, respectively.

Grain sizes were determined by statistical analysis of SEM. The previous studies [27,28,31,46] have confirmed that the contribution of grain boundaries to the strength in accordance with the Hall–Petch relationship. The boundary strengthening was calculated as equation:

(4) $$\sigma_{GB}=K_{HP}/{\left(D_{av}\right)}^{1/2}$$

where the Hall–Petch strengthening coefficient (K_HP_) is defined as the slope of the linear relationship between flow stress and the inverse square root of grain size for polycrystalline metals. D_av_ is the average grain size. Studies have reported K_HP_ values varying from 120 MPa·μm^1/2^ to 700 MPa·μm^1/2^ [30,33,35,36,37,38,39]. In this study, the value of 400 MPa·μm^1/2^ is adopted [33,37,38]. Microstructural parameters for strengthening calculation were shown in Table 3. It is critical to emphasize that K_HP_ exclusively quantifies the grain boundary strengthening contribution and does not include the role of dislocation strengthening and other factors in grains. The results are 176.2 MPa and 212.9 MPa for Sample A and Sample B, respectively.

The solid solution concentrations of Ti and Nb in two experimental steels after 620 °C coiling process were determined according to the solubility product combined with the optimum chemical ratio [37]. The results reveal that almost all these elements precipitate as TiC/NbC, and their solid solution content can be ignored. In addition to TiC/NbC, the C element in steel almost exists in the form of Fe_3_C particles during the 620 °C coiling process; the strengthening contribution of TiC/NbC and Fe_3_C is mainly precipitation strengthening [33]. The strengthening contribution of other alloying elements to the strength of steel is attributed to solid solution strengthening. Strengthening calculated based on the chemical composition provided in Table 2. Solid solution strengthening is typically quantified using the relationship:

(5) $$\sigma_{ss}=\sum_{i=1}^{n}K_{i}C_{i}$$

where K_i_ represents the strengthening coefficient for each solute element and C_i_ represents the concentration of the elements [47]. The results are 30.5 MPa and 63.8 MPa for Sample A and Sample B, respectively.

**Figure 10:**
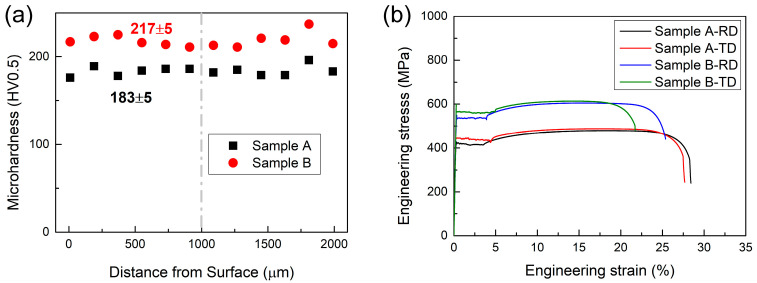
Mechanical properties of the two experimental steels: (**a**) micro-hardness distribution curves along the thickness of the samples, (**b**) engineering stress–strain curves. The grey dashed line represents the middle position in the thickness direction of the sample.

Based on the model between quantitative microstructural parameters and strength, the flow stress at (*σ*_s_) consists of five parts, namely lattice friction stress *σ*_0_, solid-solution strengthening σ_ss_, dislocation strengthening *σ*_dis_, boundary strengthening *σ*_GB_, and particle strengthening *σ*_P_. The flow stress *σ*_s_ was calculated utilizing linearly addition of the five parts:

(6) $$\sigma_{s}=\sigma_{0}+\sigma_{ss}+\sigma_{dis}+\sigma_{GB}+\sigma_{P}$$

where σ_0_ is the friction stress, taken to be 50 MPa for bcc Fe [30]. Since there is a strong correlation between microstructure and mechanical properties, it is essential to systematically test mechanical properties and analyze the influence of microstructure on these properties. Figure 10a illustrates the microhardness distribution along the thickness direction for both steels, revealing good uniformity in the two steels with no significant differences in hardness between the surface and center regions. The tensile curves in Figure 10b demonstrate that Sample B exhibits significantly higher tensile stress (R_m_) and yield stress (R_p0.2_) compared to Sample A. Furthermore, the minimal variation in mechanical properties between the RD and TD indicates that the current texture composition (dominated by α-fiber and γ-fiber textures with minor θ-fiber texture) does not induce significant mechanical anisotropy. Detailed mechanical property parameters of the experimental steels are summarized in Table 4.

**Figure 11:**
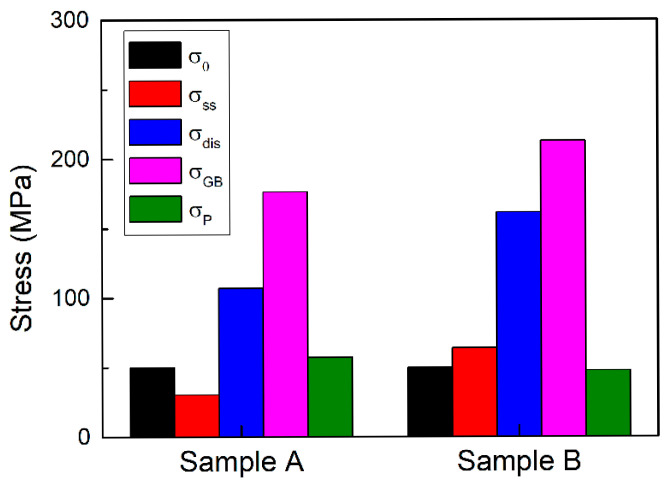
Histogram showing contributions of five parts, ranked accordingly.

Through comprehensive analysis combining calculated strengths and experimental tensile testing data, the contribution of five strengthening mechanisms were quantified (as shown in Figure 11). The study revealed that among the five mechanisms, grain boundary strengthening dominates, while dislocation strengthening ranks second with a still significant contribution. In fact, grain refinement strengthening and dislocation strengthening are extremely important strengthening mechanisms in many steel materials and even in other materials, such as tantalum alloys [30,32,33,34,45]. However, changes in processing conditions can cause alterations in the ranking of these strengthening mechanisms. For instance, Xu et al. [45] demonstrated that increasing the aging process can enhance the strength of As-Coiled samples by 60 MPa owing to the introduction of clusters. Ma et al. [32] revealed that lattice friction stress is the dominant factor for strengthening under low cumulative strains, whereas grain boundary and dislocation strengthening play a leading role under high cumulative strains. Therefore, it is both necessary and essential to conduct a systematic analysis of the strengthening contributions for each material based on its specific processing techniques and chemical composition.

Compared to conventional C-Mn steels, the key advantage of HSLA steels lies in the introduction of microalloying elements, which provide substantial strength increments with particle strengthening. These indicate that the primary strengthening mechanisms in automotive HSLA steels are grain refinement strengthening and dislocation strengthening, with further mechanical properties enhancement critically dependent on the precise addition of microalloying elements and synergistic control of heat treatment processes. Hot rolling parameters (e.g., finish rolling temperature, cooling rate, and coiling temperature) govern the evolution of grain size. The higher alloy content (particularly carbon, such as Sample B) significantly improves mechanical properties during hot deformation and subsequent phase transformations through the following mechanisms: Alloying elements segregate at grain boundaries, forming a solute drag effect that hinders the migration of grain boundaries, thereby refining grains. The precipitates (e.g., Fe_3_C, NbC and TiC) formed by carbon and microalloying elements pin dislocations, hindering dislocation rearrangement and annihilation, thus maintaining high dislocation density. By implementing multi-scale microstructural optimization (grain size, dislocation density and precipitate distribution) and quantifying the microstructural parameters, the design philosophy for optimizing HSLA steel performance can be realized. In our current work, precipitation strengthening was calculated by subtracting the calculated values of other factors from the tensile test values. If it is necessary to delve into the size distribution of precipitates and understand their contribution within the microstructure, conducting specific analyses that focus on the precipitate volume fraction and the average size of precipitates is indeed crucial. To calculate the effect of precipitation strengthening, we will rely on the Ashby-Orowan equation. This framework correlating microstructural parameters and mechanical properties through a synergistic combination of physical metallurgy principles, providing a quantitative basis for processing parameter selection in the development of higher-grade HSLA steels in the future.

## 4. Conclusions

The microstructure and mechanical properties of two types of automotive HSLA steels with different strength grades were investigated. The following conclusions were drawn.

(1) The microstructure of both steels comprises predominantly ferrite along with spheroidized carbides/pearlites. Sample A displays equiaxed ferrite grains with localized pearlite colonies, while Sample B forms pronounced elongated morphology and formed a typical band structure.
(2) The ultimate tensile stress and yield stress of the Sample B are higher than those of the Sample A. The texture components of both steels are dominated by α-fiber and γ-fiber textures, with minor θ-fiber texture, which does not induce significant mechanical anisotropy, resulting in minimal variation in mechanical properties between the RD and TD.
(3) Based on measured microstructural parameters and experimental tensile testing data, the contribution of five strengthening mechanisms was quantified. Grain boundary strengthening dominates, while dislocation strnengthening ranks second with a still significant contribution.

## Figures and Tables

**Table 1:** CSP rolling parameters of the two experimental steels.

Samples	Finisher Entry Temperature/°C	Finisher Delivery Temperature/°C	Coiling Temperature/°C	Sheet Thicknesses/mm
A	1200	890	620	2
B	1245	890	620	2

**Table 2:** Chemical composition of the two experimental steels (wt.%).

Samples	C	Si	Mn	Al	S	P	Ti	Nb	Fe
A	0.06	0.07	0.49	0.032	0.002	0.014	0.04	0.01	Bal.
B	0.12	0.05	1.46	0.024	0.004	0.012	0.03	0.03	Bal.

**Table 3:** Microstructural parameters for strengthening calculation of the two experimental steels.

Samples	D_av_/μm	KAM_ave_/°	ρ^GND^	K_HP_/MPa·μm^1/2^	M
A	5.16 ± 2.77	0.47	1.32 × 10^14^	400	2.9
B	3.53 ± 1.65	1.07	3.01 × 10^14^	400	2.9

**Table 4:** Mechanical property parameters of the experiment steels include yield stress (R_p0.2_), ultimate tensile stress (R_m_), and total elongation (A).

Steel		HV	R_p0.2_/MPa	R_m_/MPa	A/%
Sample A	RD	183 ± 5	421 ± 2	479 ± 4	29.1 ± 1.5
	TD		442 ± 4	489 ± 4	28.3 ± 1.5
Sample B	RD	217 ± 5	536 ± 1	603 ± 3	26.0 ± 1.3
	TD		564 ± 4	615 ± 5	22.3 ± 0.3

## Data Availability

The original contributions presented in this study are included in the article. Further inquiries can be directed to the corresponding authors.
